# Cellular Zinc Homeostasis Contributes to Neuronal Differentiation in Human Induced Pluripotent Stem Cells

**DOI:** 10.1155/2016/3760702

**Published:** 2016-05-09

**Authors:** Stefanie Pfaender, Karl Föhr, Anne-Kathrin Lutz, Stefan Putz, Kevin Achberger, Leonhard Linta, Stefan Liebau, Tobias M. Boeckers, Andreas M. Grabrucker

**Affiliations:** ^1^Institute for Anatomy and Cell Biology, Ulm University, 89081 Ulm, Germany; ^2^Department of Anaesthesiology, University of Ulm, 89081 Ulm, Germany; ^3^Institute of Neuroanatomy, Eberhard Karls University of Tübingen, 72074 Tübingen, Germany; ^4^WG Molecular Analysis of Synaptopathies, Neurology Department, Neurocenter of Ulm University, 89081 Ulm, Germany

## Abstract

Disturbances in neuronal differentiation and function are an underlying factor of many brain disorders. Zinc homeostasis and signaling are important mediators for a normal brain development and function, given that zinc deficiency was shown to result in cognitive and emotional deficits in animal models that might be associated with neurodevelopmental disorders. One underlying mechanism of the observed detrimental effects of zinc deficiency on the brain might be impaired proliferation and differentiation of stem cells participating in neurogenesis. Thus, to examine the molecular mechanisms regulating zinc metabolism and signaling in differentiating neurons, using a protocol for motor neuron differentiation, we characterized the expression of zinc homeostasis genes during neurogenesis using human induced pluripotent stem cells (hiPSCs) and evaluated the influence of altered zinc levels on the expression of zinc homeostasis genes, cell survival, cell fate, and neuronal function. Our results show that zinc transporters are highly regulated genes during neuronal differentiation and that low zinc levels are associated with decreased cell survival, altered neuronal differentiation, and, in particular, synaptic function. We conclude that zinc deficiency in a critical time window during brain development might influence brain function by modulating neuronal differentiation.

## 1. Introduction

Zinc is an essential trace metal interacting with a plethora of proteins. It plays a functional role in structural, regulatory, and signaling processes and thus is essential for a healthy brain. However, abnormally high levels of zinc are cytotoxic. Therefore, zinc levels have to be highly regulated during embryogenesis and development of the central nervous system (CNS). It is thus not surprising that zinc deficiencies can contribute to the occurrence of numerous human birth defects involving CNS malformation [1, 2]. On a mechanistic point of view, zinc has many roles in the developing and adult brain [3]. For example, zinc is an essential catalytic component of many different mammalian enzymes, such as DNA and RNA polymerases and histone deacetylases [4] needed for DNA replication and cellular proliferation. Additionally, zinc-dependent enzymes such as metalloproteinases and zinc-binding proteins such as metallothioneins (MTs) have a function in metabolism and zinc signaling [5]. Furthermore, many protein-protein interactions and DNA-binding properties of receptors [6] and transcription factors known to regulate key genes involved in cellular proliferation and neurogenesis are mediated by zinc-finger motifs [7, 8]. Intriguingly, maternal zinc deficiency has been identified as a risk factor for the development of autism in the offspring [9]. Further, mice exposed to zinc deficiency during brain development display autism like behavior later in life [10, 11]. Therefore, zinc signaling might play a crucial role during brain development, in particular neurogenesis and synaptogenesis, and by that ultimately mediate correct circuit formation.

Cellular zinc homeostasis is regulated by transporters, such as DMTs (divalent metal transporters), ZnTs (zinc transporters of the SLC30A family), and ZIP (Zrt-Irt-like proteins of the SLC39A family), and intracellular zinc-binding proteins, in particular metallothioneins (MTs). Transmembrane transporters mediate the uptake and removal of zinc and transport of zinc into and out of intracellular organelles. ZnT proteins transport zinc out of the cytosol and ZIP proteins move zinc into the cytosol. Zinc binding in the cytosol is mostly regulated by proteins of the MT family (MT-1, MT-2, and MT-3), which bind zinc transiently and are therefore able to provide zinc for signaling processes [12, 13].

It was reported that zinc may play a role in the control of both developmental and adult neurogenesis mediated by proliferating adult stem cells in the subgranular zone of the dentate gyrus [14]. However, on a cellular level, the underlying mechanisms that regulate zinc homeostasis in differentiating neurons and the influence of different zinc levels on differentiation efficacy and nerve cell function after differentiation are so far not well understood.

Here, we used human induced pluripotent stem cells (hiPSC) as model system for neuronal differentiation to determine the cellular consequences of altered zinc levels. To that end we used iPS cells from keratinocytes of two healthy controls [15]. iPS cells are somatic cells that can be reprogrammed to a pluripotent state by gene transfer [16–18]. As pluripotent stem cells, they can be differentiated into several lineages, of which we choose a neuronal fate. We differentiated iPS cells into neuronal precursor cells (NPCs) and neurons using conditions that favor the generation of motor neurons and evaluated the differential expression of zinc homeostasis genes and outcomes of altered zinc levels.

## 2. Methods

### 2.1. Materials

DMEM/F12 + GlutaMAX, DPBS without Ca^2+^/Mg^2+^, GlutaMAX, NEAA antibiotic-antimycotic, natural essential amino acids, knockout serum replacement, BDNF, GDNF, IGF-1, B27, FBS, and N2 were purchased from Gibco/Life Technologies. DPBS with Ca^2+^/Mg^2+^ was obtained from PAA. mTeSR1 stem cell medium and dispase were purchased from Stemcell Technologies. Purmorphamine was obtained from Calbiochem. Insulin was obtained from SAFC. ROCK inhibitor was purchased from Ascent Scientific and *β*-mercaptoethanol was purchased from Millipore. HESC qualified matrigel was obtained from BD Biosciences. Retinoic acid, poly-L-ornithine, laminin, heparin sodium salt, sodium selenite, apotransferrin, putrescin, progesterone, acetylcholine, glutamate, GABA, glycine, and NMDA were purchased from Sigma-Aldrich. Cyclothiazide was obtained from Tocris. Ultra-low attachment flasks were purchased from Corning Costar and *μ*-dishes (35 mm, low) were purchased from Ibidi. Chelex 100 resin was purchased from Bio-Rad. Tissue-Tek® was obtained from Sakura. The Apoptosis/Necrosis/Healthy Cell Detection kit was purchased from PromoKine and ApoTox-Glo Triplex Assay was purchased from Promega. Triton X-100 was procured from Roche. RNeasy Mini and Micro kit, Quantitect primers, QuantiFast SYBR Green RT-PCR kit, RT^2^ First Strand kit, and customized RT^2^ profiler rotor-disks were obtained from Qiagen. Primary antibodies were purchased from Synaptic Systems (Homer1 1 : 500, Synaptophysin 1 : 500, and GRIA3 1 : 500), Abgent (CHRNA3 1 : 500), UC Davis/NIH NeuroMab Facility (GABA-A-R*α*1 1 : 500), Covance (SMI-32 (NEFH) 1 : 1000), R&D Systems (active caspase-3 1 : 1000), and Sigma-Aldrich (NMDAR1 1 : 500). Alexa Fluor-conjugated secondary antibodies were purchased from Invitrogen (1 : 1000). Unless otherwise indicated, all other chemicals were obtained from Sigma-Aldrich.

### 2.2. Cell Culture

IPS cell lines were generated by Linta et al. as previously described [15]. iPS cells were cultured in mTeSR1 medium at 37°C, 5% O_2_, and 5% CO_2_. Differentiation into motor neurons was performed as previously described by Hu and Zhang [19]. For the formation of embryoid bodies (EB), iPS cells were cultured in suspension in hESC medium (DMEM/F12 + 20% knockout serum replacement + 1% NEAA + 1%  *β*-mercaptoethanol + 1% antibiotic-antimycotic) in ultra-low attachment flasks (Corning Costar). ROCK inhibitor was added for the first 48 h. Neurodifferentiation was induced by changing the medium to (DMEM/F12 + 24 nM sodium selenite + 16 nM progesterone + 0.08 mg/mL apotransferrin + 0.02 mg/mL insulin + 7.72 *μ*g/mL putrescin + 1% NEAA, 1% antibiotic-antimycotic + 50 mg/mL heparin + 10 *μ*g/mL of the neurotrophic factors BDNF, GDNF, and IGF-1) at e03. At e07 EB were plated on laminin coated 12-well plates. 0.1 *μ*M of retinoic acid (RA) was added at e09. At e14 neural rosettes (NR) were detached and cultured in suspension with addition of 1 *μ*M purmorphamine. 2-3 neural stem cell (NSC) spheres were plated on a 35 mm *μ*-dish (Ibidi) coated with poly-L-ornithine (PLO) and laminin. Retinoid acid was reduced to 0.05 *μ*M. Medium was changed 1x/week. Motor neurons were analyzed at d21 and d42 after final plating.

### 2.3. Establishment of Different Zinc Conditions

Zinc deficient medium was generated by depletion of all divalent cations using Chelex 100 resin (Bio-Rad) as described by the manufacturer and as previously published [20]. To that end mTeSR1, hESC medium, and basal differentiation medium were incubated with Chelex 100 beads that were subsequently removed by centrifugation, a cell strainer, and sterile filtering with 0.2 *μ*m sterile filters. Original cation concentrations were reestablished for all divalent cations present in neurobasal medium (0.247 *μ*M Fe_2_(NO_3_)_3_, 0.81 mM MgCl_2_, and 1.8 mM CaCl_2_) except for ZnSO_4_·4H_2_O. The original pH of the medium was readjusted with HCl (mTeSR1 pH 8.2; hESC basal pH 8.0; basal differentiation medium pH 7.5). Supplemental factors were added to the zinc depleted medium right before use. As a control condition, the original zinc concentration of zinc depleted medium was reestablished. Zinc supplemented medium was prepared by addition of 10 *μ*M ZnCl_2_ to the original medium.

### 2.4. Preparation of Cryosections of NSC Spheres

NSC spheres were fixed with 4% paraformaldehyde/10% saccharose and washed three times with PBS and resuspended in 100 *μ*L PBS. Tissue-Tek in small aluminium foil containers was snap-frozen until it was viscous. NSC spheres were transferred to the viscous Tissue-Tek and snap-frozen until everything was solid. 30 *μ*m cryosections were made at the cryostat. Alternatively, NSC were live-stained using the Apoptosis/Necrosis/Healthy Cell Detection kit (PromoKine) according to the manufacturer's instructions for suspension cells before fixing. Staining time was enlarged to 45 min to ensure penetration of NSC spheres. Fixing and washing were performed in buffers containing 1.25 mM calcium-chloride to ensure annexin V binding to phosphatidylserine.

### 2.5. Immunocytochemistry

For immunofluorescence, the cultures were fixed with 4% paraformaldehyde/4% sucrose/PBS at 4°C for 20 min and processed for immunocytochemistry. After washing 3 × 5 min with 1x PBS, cells were permeabilized with 0.2% Triton X-100 for 5 min at RT. Blocking was performed with 5% FBS in 1x PBS for 1 h at RT, followed by primary antibody incubation with at 4°C overnight. After a 3 × 5 min washing-step with 1x PBS, incubation with the Alexa Fluor-coupled secondary antibody followed for 1 h. Cells were mounted with ProLong® Gold antifade reagent either with or without DAPI.

### 2.6. Metabolic Apoptosis Assay

Embryoid bodies (EB) grown in zinc depleted or zinc replete conditions were harvested and transferred to an opaque 96-well plate in different dilutions with zinc depleted or zinc replete DMEM/F12 + 24 nM sodium selenite + 16 nM progesterone + 0.08 mg/mL apotransferrin + 0.02 mg/mL insulin + 7.72 *μ*g/mL putrescin + 1% NEAA, 1% antibiotic-antimycotic + 50 mg/mL heparin + 10 *μ*g/mL of the neurotrophic factors BDNF, GDNF, and IGF-1 in a final volume of 100 *μ*L/well. Ethanol-treated EB from the zinc repletion condition were used as a positive control, and medium without cells was used as a blank. The ApoTox-Glo Triplex Assay (Promega) was performed as described in the manufacturer's protocol. 20 *μ*L of combined substrates cell-permeant GF-AFC and cell-impermeant bis-AAF-R110 in assay buffer was added to the cell solution and incubated for 45 min at 37°C. Fluorescence was read in a PerkinElmer 2030 Explorer at 405 nm (excitation) and 460 nm (emission) for viability and 485 nm (excitation) and 535 nm (emission) for cytotoxicity. 100 *μ*L of Caspase-Glo® 3/7 Substrate in Caspase-Glo® 3/7 Buffer was added to the wells and incubated at 37°C for 2 h. Luminescence was read in a PerkinElmer 2030 Explorer. Viability/cytotoxicity ratios were not significantly different. Apoptotic cells were normalized to number of viable cells.

### 2.7. Quantitative Real-Time PCR

Isolation of total RNA from iPS cells in different stages of motor neuron differentiation was performed using the RNeasy Mini kit (Qiagen) as described by the manufacturer including all additional purification steps. For the reverse transcriptase-mediated PCR studies, first strand synthesis and real-time quantitative RT-PCR amplification were carried out in a one-step, single-tube format using the QuantiFast SYBR Green RT-PCR kit. Thermal cycling and fluorescent detection were performed using the Rotor-Gene-Q real-time PCR machine (model 2-Plex HRM) (Qiagen). The qRT-PCR was assayed in 0.1 mL strip tubes in a total volume of 20 *μ*L reaction mixture containing 1 *μ*L of undiluted total RNA, 2 *μ*L of QuantiTect Primer Assay oligonucleotides, 10 *μ*L of 2x QuantiFast SYBR Green RT-PCR Master Mix supplemented with ROX (5-carboxy-X-rhodamine) dye, and 6.8 *μ*L of RNase-free water and 0.2 *μ*L of QuantiFast RT Mix. Amplification conditions were as follows: 10 min at 55°C and 5 min at 95°C, followed by 40 cycles of PCR for 5 s at 95°C for denaturation and 10 s at 60°C for annealing and elongation (one-step). The SYBR Green I reporter dye signal was measured against the internal passive reference dye (ROX) to normalize non-PCR-related fluctuations. Resulting data were analyzed utilizing the hydroxymethylbilane synthase (HMBS) gene as an internal standard to normalize transcript levels through all stages of differentiation. Cycle threshold (ct) values were calculated by the Rotor-Gene Q software (version 2.0.2). All qRT-PCR reactions were run in biological triplicate for each cell line. To ascertain primer specificity a melting curve was obtained for the amplicon products to determine their melting temperatures. Real-time quantitative PCR was carried out using oligonucleotides allowing to investigate expression of NEFH as well as MT2A, MT3, MTF1, ProSAP1/Shank2, ProSAP2/Shank3, Shank1, ZnT1, ZnT2, ZnT3, ZnT4, ZnT5, ZnT6, ZIP1, and ZIP3 (validated primer pairs, Quantitect primer assay, Qiagen). The set of zinc homeostasis and other zinc-related genes included in the analyses of this study was chosen based on a literature search for reports on the expression and function of specific zinc transporters and zinc-binding genes in the brain and spinal cord [21–25].

For high-throughput screening of synaptic genes total RNA was isolated using the RNeasy Mini kit according to the manufacturer's manual. All additional purification steps were performed and RNA eluted in a total of 15 *μ*L RNAse-free water. Genomic DNA elimination and cDNA synthesis were performed with 800 ng RNA using the RT^2^ First Strand Kit (Qiagen) as described by the manufacturer. Quantitative real-time PCR screening was performed using the RT^2^ SYBR Green qPCR Mastermix and customized RT^2^ profiler rotor-disks with preimmobilized primers (Qiagen #CAPA9600-12:CAPH13069R) according to the manufacturer's protocol in a reaction volume of 20 *μ*L. Ct values were calculated by the Rotor-Gene Q software as previously described and normalized to NEFH as an internal standard for mature motor neurons.

### 2.8. Microscopy

Fluorescence images were obtained with an upright Axioscope microscope equipped with a Zeiss CCD camera (16 bits; 1280 × 1024 ppi) using Axiovision software (Zeiss) and analyzed using Axiovision and ImageJ (v1.49o) software.

### 2.9. Electrophysiology

Single cell patch-clamp electrophysiology of motor neurons d42 after plating was performed using intracellular buffer (140 mM KCl, 2 mM MgCl_2_, 4 mM EGTA, 4 mM ATP·2Na, and 10 mM HEPES; pH 7.2) and extracellular buffer (145 mM NaCl, 5 mM KCl, 1 mM MgCl_2_, 1.5 mM CaCl_2_, 6 mM glucose, and 12 mM HEPES; pH 7.4). Standard parameters like resting membrane potential, membrane resistance, serial resistance, cell size (capacitance using the *c*-slow compensation method and the “sine + dc” method according to Lindau and Neher [26]), synaptic currents (voltage clamp mode), synaptic events (current clamp mode), and half-width as well as peak height after AP induction were assessed. In addition cells were stimulated either with 100 *μ*M acetylcholine, 500 *μ*M glutamate, 10 *μ*M GABA, and 500 *μ*M glutamate along with 100 *μ*M cyclothiazide or with 50 *μ*M NMDA along with 10 *μ*M glycine (switch to Mg free buffer).

### 2.10. Statistics

Statistical analysis was performed using Microsoft Excel, GraphPad Prism 5, and SPSS with a significance level set to 0.05 (^*∗*^ *p* ≤ 0.05;  ^*∗∗*^ *p* ≤ 0.01;  ^*∗∗∗*^ *p* ≤ 0.001). According to the parameters tested, values were analyzed for normal distribution and Student's *t*-test or 1-way ANOVA with subsequent post hoc analysis (Tukey's multiple comparison test) was performed. Only in case no iPSC line specific differences were detected, values from both lines were pooled.

## 3. Results

### 3.1. Expression of Zinc Homeostasis Genes during Stem Cell Differentiation into Motor Neurons

**Figure 1:**
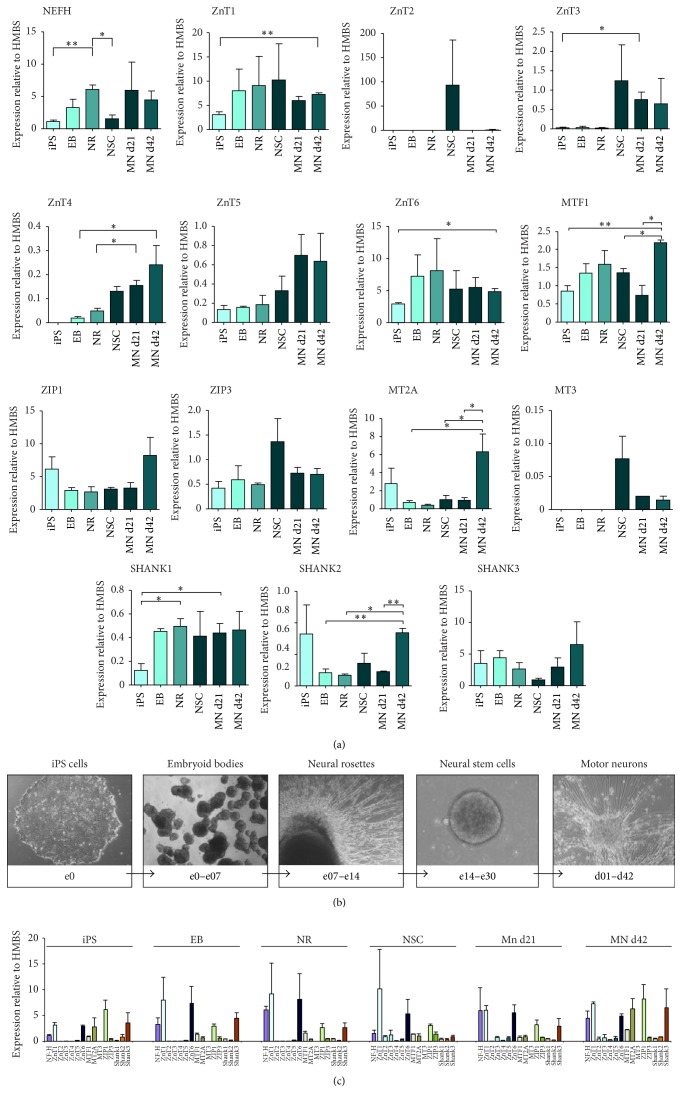
Expression of zinc homeostasis genes during stem cell differentiation into motor neurons. (a) Quantitative evaluation of mRNA expression levels normalized to HMBS of selected zinc homeostasis and the zinc-dependent SHANK genes. Analyses were performed in triplicate (*n* = 3) and the mean normalized expression is shown. Neurofilament H (NEFH) expression was used to control successful differentiation and increases significantly in NRs and rises again after a significant decrease in NSC in suspension (iPSC versus NR: *p* = 0.0028; NR versus NSC: *p* = 0.0175). Several zinc transporters show developmental stage-dependent expression as well as altered expression levels during differentiation. Expression of ZnT1 is significantly increased in motor neurons at d42 compared to the iPS cell stage (*p* = 0.0086). ZnT2 expression in turn was only detected above background in NSC. Expression of ZnT3 is very low in early stages (iPS, EB, and NR) but increases in NSC and becomes significant in motor neurons (iPSC versus MN d21: *p* = 0.0239) in which ZnT3 levels remain elevated. No expression of ZnT4 was detected in iPS cells, but mRNA levels gradually increase from EB to motor neurons (EB versus MN d42: *p* = 0.0352; NR versus MN d21: *p* = 0.0384). This is also observed for ZnT5 expression; however it is only seen as trend. Additionally, the expression of ZnT6 was found to be significantly higher in motor neurons compared to iPS cells (iPS versus MN d42: *p* = 0.0178). Along with the increase in expression of some zinc transporters during motor neuron differentiation, the mRNA levels of MTF1 are significantly higher in motor neurons compared to iPS cells (iPSC versus MN d42: *p* = 0.0074; NSC versus MN d42: *p* = 0.0170; MN d21 versus MN d42: *p* = 0.0263). While ZIP1 and ZIP3 are expressed on similar level through all stages of motor neuron differentiation, the expression of MT2A shows some variance in iPSC but otherwise is significantly higher in motor neurons compared to most previous stages and significantly increases in motor neurons between d21 and d42 (EB versus MN d42: *p* = 0.0347; NSC versus MN d42: *p* = 0.0474; MN d21 versus MN d42: *p* = 0.0397). The expression of MT3 in turn could only be detected in NSC and motor neurons but not in earlier stages. Although SHANK proteins are associated with synapses that only occur in motor neurons, their expression can be already detected in iPS cells and later stages of motor neuron differentiation. SHANK1 and SHANK2 expression increases towards later stages (SHANK1: iPSC versus NR: *p* = 0.0225; iPSC versus MN d21: *p* = 0.0327) (SHANK2: EB versus MN d42: *p* = 0.0061; NR versus MN d42: *p* = 0.0132; MN d21 versus MN d42: *p* = 0.0017), although the expression of SHANK2 in iPS cells shows high variability. SHANK3 is expressed on similar level throughout differentiation. (b) Representative images of hiPSC undergoing differentiation to motor neurons (MN) via the generation of embryoid bodies (EB), neural rosettes (NR), and neural stem cells (NSC). (c) Comparison of mRNA expression levels across different zinc homeostasis genes. In general, expression levels of ZnT1 and ZnT6 were the highest compared to other ZnTs across all developmental stages. Similarly, ZIP1 expression was high throughout all phases of motor neuron differentiation, while MTF1 and MT2A expression increased in old motor neurons. ^*∗*^ *p* ≤ 0.05; ^*∗∗*^ *p* ≤ 0.01.

iPS cells from two control cell lines were differentiated to motor neurons using a well-characterized protocol [19, 27] (Figure 1(a)). Identity of motor neurons was confirmed by the motor neuronal marker NEFH. To investigate whether specific zinc homeostasis genes are developmentally regulated, we determined the mRNA expression levels of selected zinc homeostasis genes and the zinc-dependent SHANK genes that have been associated with autism spectrum disorders (ASD) [28] during neuronal differentiation in various stages (iPS cells, embryoid bodies, neural rosettes, neural stem cells, and motor neurons) (Figure 1(b)). Several zinc transporters show developmental stage-dependent expression as well as altered expression levels during differentiation. While ZIP1 and ZIP3 are expressed on similar level throughout all stages of motor neuron differentiation, the expression of transporters from the ZnT family shows more variations. In particular, an increase in ZnT4 expression from iPS cells to motor neurons can be detected. In addition, the expression of ZnT1 is significantly increased in motor neurons at d42 compared to the iPS cell stage. The expression of ZnT3 is very low in early stages but also significantly increases in immature motor neurons and remains elevated in mature motor neurons. Similarly, no expression of ZnT4 was detected in iPS cells, but mRNA levels gradually increase from EB to motor neurons, which can also be observed for ZnT5 expression. Additionally, mRNA levels of ZnT6 were found to be significantly higher in motor neurons compared to iPS cells. In contrast, ZnT2 expression was only detected in NSC (Figure 1(b)).

Zinc homeostasis is regulated not only by the influx and efflux of zinc via transporters, but also by buffering of zinc due to zinc-binding proteins. Metallothioneins (MTs) thereby play a crucial role. The expression of MTs is regulated in response to the zinc levels present inside cells. This process is mediated by the transcription factor MTF1. Interestingly, the mRNA level of MTF1 itself is significantly higher in motor neurons compared to iPS cells.

The expression of MT2A, apart from some variance in iPS cells, is low during all stages of motor neuron development compared to adult motor neurons at d42. Thus, MT2A expression very specifically significantly increases in motor neurons between d21 and d42. The expression of MT3 in turn was only detected in NSC and motor neurons but not in earlier stages, which is in line with the CNS specific expression pattern of MT3 [29]. No significant difference was detected between young and old motor neurons (Figure 1(b)). Finally, proteins of the SHANK family have been shown to bind zinc (Shank2 and Shank3) [10, 30, 31] and are key proteins of synapses in the CNS and also occur at neuromuscular junctions [32]. Interestingly, although SHANK proteins are associated with synapses that only occur in differentiated motor neurons, their expression can be already detected in iPS cells and later stages of motor neuron differentiation, hinting towards an additional role outside postsynaptic densities (PSDs) [33]. Particularly SHANK3 seems to be expressed on similar level throughout differentiation. In contrast, although also expressed in each stage, mRNA levels of SHANK1 and SHANK2 seem to increase towards later stages (Figure 1(b)).

Comparing not only the expression of a single gene between different developmental stages but also the general expression levels between genes (Figure 1(c)), expression levels of ZnT1 and ZnT6 were the highest compared to other ZnTs across all stages of motor neuron differentiation. Similarly, ZIP1 expression was comparably high throughout all phases of differentiation. Zinc supplementation (10 *μ*M ZnCl_2_ during all stages) does not significantly alter the expression of the aforementioned genes compared to untreated controls (see Figure S1 in Supplementary Material available online at http://dx.doi.org/10.1155/2016/3760702) with the exception of MT2A in old motor neurons.

### 3.2. Zinc Deficiency Affects Expression of Zinc Homeostasis Genes during Stem Cell Differentiation into Motor Neurons

**Figure 2:**
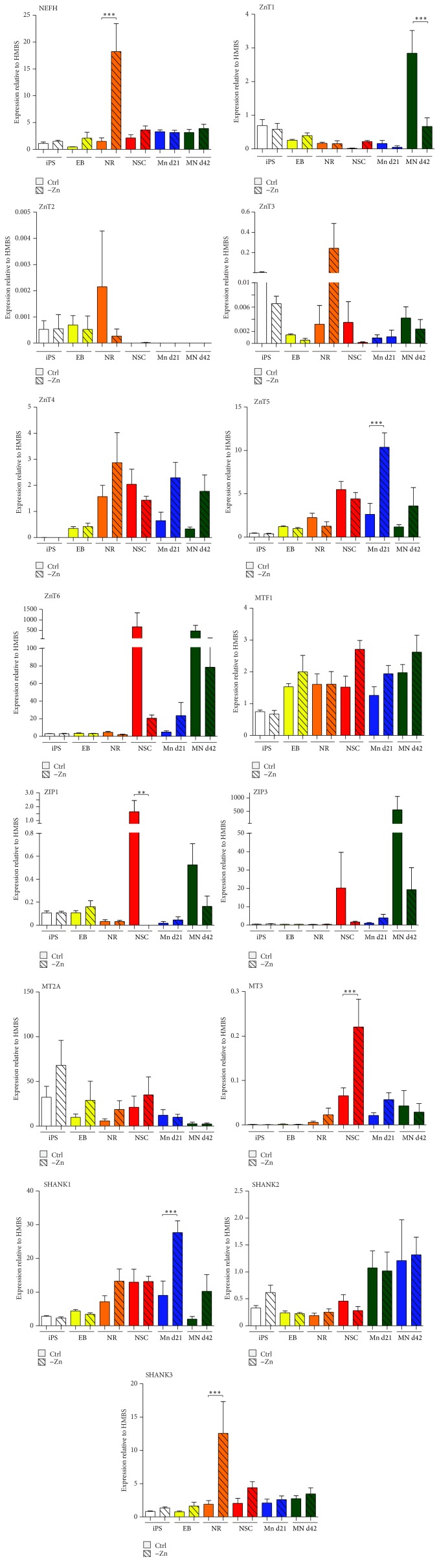
Zinc deficiency alters the expression of zinc homeostasis genes during stem cell differentiation into motor neurons. Quantitative evaluation of mRNA expression levels normalized to HMBS of selected zinc homeostasis and the zinc-dependent SHANK genes. Data shows the average normalized gene expression based on *n* = 6 measurements. Neurofilament H (NEFH) expression was used to control successful differentiation. For statistical analysis, a 1-way ANOVA was used followed by Tukey's multiple comparison test. Several zinc transporters show expression levels depending on the availability of zinc in the medium. Comparing cells grown in zinc depleted medium with cells grown in medium which was resupplemented with zinc in the amount used in control medium after zinc depletion, a significant difference in ZnT1 expression was found in d42 motor neurons (one-way ANOVA: *p* < 0.0001; MN d42 Ctrl versus −Zn: *p* < 0.05). No significant difference was detected regarding the expression of ZnT2, ZnT3, ZnT4, ZnT6, and MTF1 comparing controls and zinc deficient conditions. The expression of ZnT5 was significantly increased in motor neurons at d21 under zinc deficient conditions (one-way ANOVA: *p* < 0.0001; MN d21 Ctrl versus −Zn: *p* < 0.05). The expression of ZIP1 was significantly reduced in neuronal stem cells under zinc deficient conditions (one-way ANOVA: *p* = 0.0012; NSC Ctrl versus −Zn: *p* < 0.05). The expression of ZIP3 was not found to be altered, similar to the expression levels of MT2A. MT3 expression was significantly higher in neuronal stem cells under zinc deficient conditions (one-way ANOVA: *p* < 0.0001; NSC Ctrl versus −Zn: *p* < 0.05). The expression of SHANK1 was increased in motor neurons at d21 under zinc deficient conditions (one-way ANOVA: *p* < 0.0001; MN d21 Ctrl versus −Zn: *p* < 0.05) and the expression of SHANK3 in neural rosettes under zinc deficient conditions (one-way ANOVA: *p* < 0.0001; NR Ctrl versus −Zn: *p* < 0.05). No change was observed for SHANK2 expression. ^*∗∗*^ *p* ≤ 0.01; ^*∗∗∗*^ *p* ≤ 0.001.

Given that, under physiological conditions, the blood brain barrier (BBB) tightly regulates brain zinc levels protecting the brain from abnormally high zinc concentrations, zinc deficiency might likely occur during pregnancy [34], leading to alterations in brain development of the offspring [35, 36]. Thus, in the next set of experiments, we compared expression levels of the zinc homeostasis genes previously investigated under zinc depleted and zinc replete (control) conditions. To that end cells were exposed to medium that was previously zinc depleted using Chelex-100 beads or zinc replete medium as a control condition throughout all stages of neurodifferentiation (Figure 2). Our results show that zinc deficiency leads to gene and differentiation stage-dependent changes. Zinc deficiency leads to a decrease of ZnT1 expression in motor neurons at d42, and a significant upregulation of ZnT5 expression in motor neurons at d21 and less so at d42. The expression of ZIP1 was significantly decreased in the NSC stage under zinc deficient conditions, while the expression of MT3 significantly increased. Again expression levels of SHANK proteins were sensitive for altered zinc concentrations in the medium. The expression of SHANK1 significantly increased in motor neurons at d21 and of SHANK3 in neural rosettes under zinc deficient conditions.

### 3.3. Zinc Deficiency Significantly Impairs iPS Cell Differentiation

**Figure 3:**
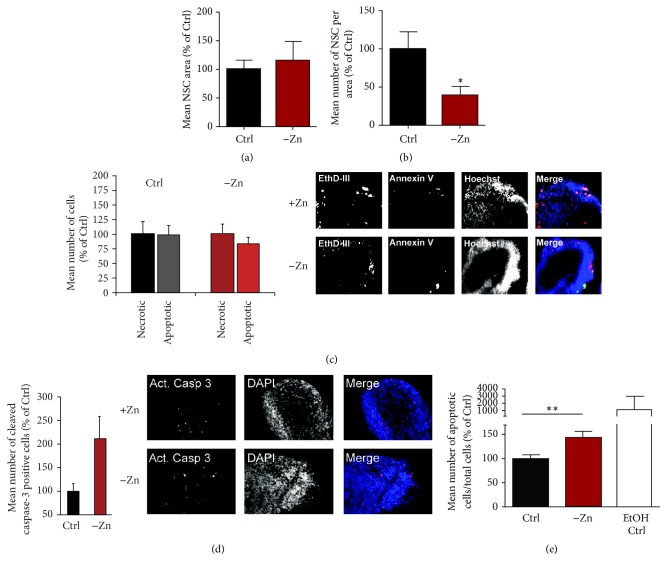
Zinc deficiency significantly impairs iPS cell differentiation. (a) The average size of NSC clusters is not significantly altered upon zinc deficiency. (b) The mean number of NSC clusters per area measured on a 10 cm petri dish is significantly reduced in zinc deficient conditions (*t*-test, *p* = 0.0281; *n* = 8). (c) Apoptosis and necrosis were evaluated for the NSC stage during differentiation. No difference in the number of cells labeled with markers for apoptosis or necrosis was found between zinc deficient and zinc sufficient media (*t*-test, *n* = 20). Right panel: exemplary images of NSC stained with annexin V labeled with FITC (apoptotic cells) and ethidium homodimer III (necrotic cells) and DAPI (total number of cells). (d) The mean number of cells showing signals specific for cleaved caspase-3 was increased under zinc deficient conditions seen as trend (*t*-test, *p* = 0.09; *n* = 20). Right panel: exemplary images of NSC stained with anti-active caspase-3 antibody and DAPI. (e) A significant increase in apoptotic cells was detected in embryoid bodies grown under zinc deficient conditions using the ApoTox-Glo*™* Triplex Assay (*t*-test, *p* = 0.0098; *n* = 12). ^*∗*^ *p* ≤ 0.05; ^*∗∗*^ *p* ≤ 0.01.

To understand the biological consequences of the observed alterations, we analyzed the capacity of stem cells to undergo successful neuronal differentiation under zinc deficient conditions in neuronal stem cells (NSC) and embryoid bodies (EB) (Figure 3). While the average size of NSC clusters was not significantly altered upon zinc deficiency (Figure 3(a)), the number of NSC clusters was significantly reduced in zinc deficient conditions (Figure 3(b)) indicating a reduction in cell survival. However, we could not detect a significant increase in markers for apoptosis or necrosis at the NSC stage (Figure 3(c)). Apoptotic cells were identified using annexin V labeled with FITC, necrotic cells by ethidium homodimer III, and the total number of cells was assessed using Hoechst labeling. The analysis of the amount of cleaved caspase-3 shows an increase of cleaved caspase-3 in NSC grown under zinc deficient conditions, which is seen as a trend (Figure 3(d)). Activation of caspase-3 requires cleavage of its inactive form into activated p17 and p12 fragments, activating downstream processes of apoptosis. The amount of cleaved caspase-3 thus provides a read-out for initiated apoptosis.

Since we did not detect significantly increased cell death at the NSC stage, cell survival seems impaired already at earlier stages. This was confirmed by analysis of EB (Figure 3(e)). Here, we found a significant increase in the number of cells undergoing apoptosis in cells grown under zinc deficient conditions compared to controls.

### 3.4. Altered Zinc Levels Affect Glutamatergic Neuronal Differentiation and Neuronal Function

**Figure 4:**
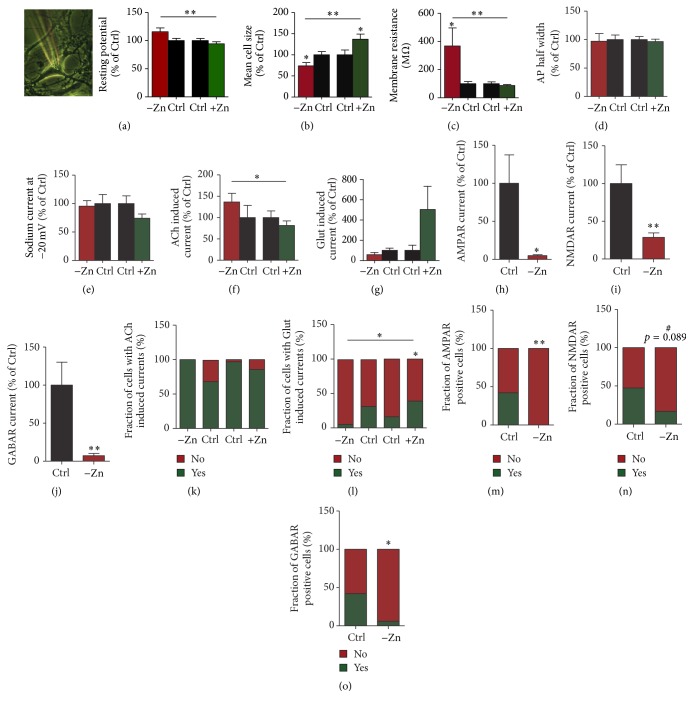
Altered zinc levels affect glutamatergic signaling. (a) Representative image of a neuron used for patch-clamp electrophysiological studies (left panel). Right panel: a significant difference in resting potentials was detected between cells grown under zinc deficient and zinc supplemented conditions (*t*-test, *p* = 0.0028; *n* = 19: Ctrl_−Zn_ and −Zn, *n* = 32: Ctrl_+Zn_, and *n* = 42: +Zn). (b) A significant difference in membrane capacity was detected between cells grown under zinc deficient and zinc supplemented conditions (*t*-test, Ctrl_−Zn_ versus  −Zn, *p* = 0.0228; Ctrl_+Zn_ versus +Zn, *p* = 0.0273; −Zn versus +Zn, *p* = 0.0016; *n* = 19: Ctrl_−Zn_ and −Zn; *n* = 32: Ctrl_+Zn_, *n* = 42: +Zn). (c) A significant difference in membrane resistance was detected between cells grown under zinc deficient and zinc supplemented conditions (*t*-test, Ctrl_−Zn_ versus  −Zn, *p* = 0.0445; −Zn versus +Zn, *p* = 0.0018; *n* = 19: Ctrl_−Zn_ and −Zn; *n* = 32: Ctrl_+Zn_; *n* = 42: +Zn). (d) Single cell patch-clamp electrophysiology did not show any significant differences in the half-width of induced action potentials (AP) and sodium currents (e). (f) A significant increase in acetylcholine (ACh) induced currents was observed in zinc deficient cells when compared to zinc supplemented cells (*t*-test, −Zn versus +Zn, *p* = 0.050; *n* = 19: Ctrl_−Zn_ and −Zn, *n* = 32: Ctrl_+Zn_, and *n* = 42: +Zn). (g) A decrease in glutamate (Glut) induced currents can be seen under zinc deficient conditions as trend. (h) AMPAR currents and NMDAR currents (i) as well as GABAR currents (j) were significantly decreased after differentiation of cells in zinc depletion conditions (*t*-test, AMPAR, *p* = 0.0186; NMDAR, *p* = 0.0099; GABAR, *p* = 0.0067; *n* = 19: Ctrl_−Zn_, *n* = 18: −Zn, *n* = 31: Ctrl_+Zn_, and *n* = 41: +Zn). (k) The number of cells showing ACh induced currents was higher in zinc deficient conditions. (l) The number of cells showing Glut induced currents was significantly less under zinc deficient conditions when compared to zinc supplemented cells (contingency (Chi square) test, *p* = 0.0113). The fraction of cells with Glut induced currents was also significantly increased in zinc supplemented cells compared to controls (contingency (Chi square) test, *p* = 0.04) (*n* = 19: Ctrl_−Zn_, *n* = 18: −Zn, *n* = 31: Ctrl_+Zn_, and *n* = 41: +Zn). (m) The fraction of cells with AMPAR currents (m) and NMDAR currents (n) as well as GABAR currents (o) was decreased after differentiation of cells in zinc depletion conditions (contingency (Chi square) test, AMPAR, *p* = 0.0031; NMDAR, *p* = 0.089; GABAR, *p* = 0.0198; *n* = 19: Ctrl_−Zn_; *n* = 17-18: −Zn). All currents were normalized to cell size.  ^#^trend (*p* value between 0.05 and 0.1). ^*∗*^ *p* ≤ 0.05; ^*∗∗*^ *p* ≤ 0.01.

Finally, to investigate whether zinc deficiency does influence not only cell survival but also the fate and developmental endpoints of cells during differentiation, we first analyzed differentiated motor neurons regarding their morphology (Figure S2) and functionality using electrophysiological read-outs (Figure 4). We stained motor neurons for the motor neuronal marker NEFH, the postsynaptic marker Homer1, and the presynaptic marker synaptophysin as well as DAPI for nuclear labeling. Morphological analysis of motor neurons derived from iPS cells grown under control and zinc supplemented conditions did not reveal any significant differences regarding soma size (Figure S2A), the number of primary dendrites (Figure S2B), and the number of synapses (Figure S2C). Using single cell patch-clamp electrophysiology, a significant difference in resting potentials was detected between cells grown under zinc deficient and zinc supplemented conditions (Figure 4(a)) as well as a significant difference in cell size assessed by capacitance measurements using the *c*-slow compensation method and the “sine + dc” method according to Lindau and Neher [26] (Figure 4(b)) and the number of ion channels active or existent as assessed by membrane resistance, both hinting towards a slightly more immature phenotype (Figure 4(c)). There was an increase in resting potential negativity in zinc depleted cells. The cells did not show any significant differences in the half-width of induced action potentials and sodium currents that could confirm a larger difference in maturity (Figures 4(d) and 4(e)).

During electrophysiological measurements, all cells were measured within the same buffer with physiological zinc concentration. We detected a significant increase in acetylcholine (ACh) induced currents in cells grown under zinc deficient conditions (Figure 4(f)). In contrast, a decrease in glutamate (Glut) induced currents can be seen under zinc deficient conditions (Figure 4(g)).

To further elucidate the alterations in glutamate signaling, we investigated AMPAR and NMDAR currents in the cells grown under different zinc levels. We observed a reduction in both AMPAR and NMDAR currents (Figures 4(h) and 4(i)) and also detected a reduction in GABAR currents (Figure 4(j)) after differentiation of cells in zinc depletion conditions. In line with these results, the number of cells showing ACh induced currents was higher in zinc deficient conditions (Figure 4(k)), while the number of cells showing Glut induced currents was significantly less when compared to zinc supplemented cells (Figure 4(l)). In addition, the fraction of cells displaying AMPAR, NMDAR (as a trend), or GABAR currents was significantly decreased in cells of the zinc depletion condition (Figures 4(m)–4(o)).

**Figure 5:**
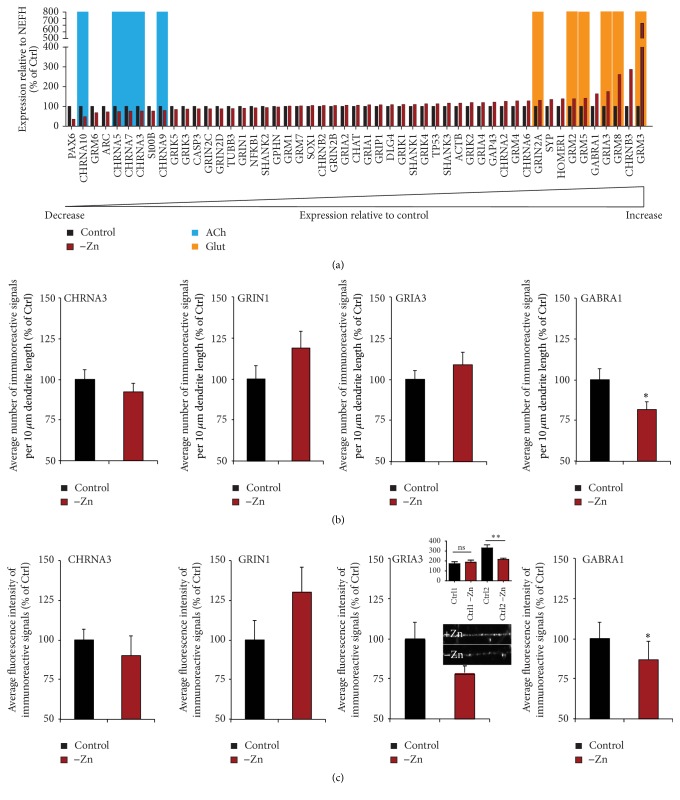
Cells differentiated under different zinc levels show altered gene expression and protein levels of neurotransmitter receptors. (a) A screening for the expression of genes associated with glutamatergic and cholinergic neurotransmission as well as synapse plasticity reveals a decrease of ACh receptors, mainly CHRNA3, CHRNA5, CHRNA7, CHRNA9, and CHRNA10, while an increase in glutamate receptor expression (GRM2, GRM3, and GRM5; GRIA3; GRIN2A) was observed. (b) Immunocytochemical analysis of *n* = 20 cells per condition. The mean number of signals per dendrite length was assessed. No differences were detected for CHRNA3, GRIN1, and GRIA3. A significant reduction was seen for GABRA1 signals (*t*-test, *p* = 0.05). (c) Immunocytochemical analysis of *n* = 20 cells per condition. The mean signal intensity per fluorescent puncta was assessed. No differences were detected for CHRNA3 and GRIN1. A significant reduction of GABRA1 signal intensity (*t*-test, *p* = 0.022) was observed. GRIA3 signal intensity was reduced only in one cell line (*t*-test, *p* = 0.0045). ns: not significant. ^*∗*^ *p* ≤ 0.05; ^*∗∗*^ *p* ≤ 0.01.

Given that the observed shift hints towards a reduction in glutamatergic and GABAergic neurotransmission favoring ACh signaling, we evaluated expression of synaptic receptors. A screening for the expression of genes associated with glutamatergic and cholinergic neurotransmission as well as synapse plasticity revealed a decrease of ACh receptors, mainly CHRNA3, CHRNA5, CHRNA7, CHRNA9, and CHRNA10, while an increase in metabotropic glutamate receptor expression (GRM2, GRM3, and GRM5) was observed (Figure 5(a)). As the alterations might reflect a compensatory mechanism in response to reduced zinc levels and are not reflected by the functional read-outs, we further investigated whether the altered gene expression is translated into changes on protein levels. To that end, we performed analyses of fluorescent signal intensity and measured the amount of immunoreactive signals after immunocytochemistry (Figures 5(b) and 5(c)). Under reduced zinc conditions, we could detect a significant decrease in the number of gamma-aminobutyric acid (GABA) receptor 1 (GABRA1) signals per dendrite and a decrease in GABRA1 fluorescent signal intensity correlating with the amount of protein present. Further, we could detect a reduction in the protein concentration of ionotropic glutamate (AMPA) receptor 3 (GRIA3), which however was only found significant in one iPSC line. No alterations were detected in GRIN1 (ionotropic glutamate (NMDA) receptor 1) and CHRNA3 (nicotinic cholinergic receptor, alpha 3).

## 4. Discussion

The global prevalence of zinc deficiency with estimated 31% [37] may have significant effects on brain development, cognition, and neurological diseases, in particular those that might be caused by an impaired glutamatergic signaling such as autism spectrum disorders [38, 39].

Here, we found that the expression of genes mediating zinc homeostasis and signaling is endogenously regulated during motor neuronal differentiation and provide a detailed data-set for gene expression of a subset of zinc transporter genes and zinc-binding proteins during stem cell differentiation into motor neurons. These regulatory processes are likely both to occur as reaction to altered demands for zinc in specific differentiation stages and to enable zinc signaling, which, for example, is involved in processes such as apoptosis. In line with this, we could show that insufficient zinc supply has an effect on cell survival and by that reduces the number of neuronal stem cells differentiating from iPSC. It has been previously reported that zinc deficiency can affect neuronal cell precursor proliferation by the induction of apoptosis via p53-mediated processes [3, 40, 41].

Given this important role of adequate zinc supply for neurogenesis, it is not surprising that physiological responses exist to tightly regulate zinc levels during neuronal differentiation. Indeed, we observed a response in gene expression of zinc homeostasis genes under zinc deficient conditions. For example, a reduction of the zinc exporter ZnT1 as observed in mature motor neurons will lead to increased retention of zinc inside the neuron. Reduced ZnT1 levels were already observed in neonatal rats exposed to zinc deficiency [42]. Similarly, reduced expression of ZnT5 in young motor neurons may reduce zinc export into cell organelles and thereby ameliorate the decrease in cytoplasmic zinc. Such a responsiveness of ZnT5 to zinc deficiency was also shown with in THP1 cells [43]. In contrast, an increase in MT3 as observed in NSC under zinc depletion might increase the ability to retain and buffer zinc inside the cell. A moderate increase of MT3 expression in response to zinc deficiency was reported before in rats [44]. However, a great variability of these alterations during the different stages of neuronal differentiation hints towards highly dynamic compensatory processes and/or further regulatory processes on protein level.

In case of an inability to compensate low zinc levels such as in our experimental conditions, not only is the number of neuronal stem cells decreased but also differentiated motor neurons show significant differences when compared to motor neurons that differentiated under zinc adequate conditions. The differences we observed cannot be explained by alterations of NMDAR, AMPAR, GABAR, or acetylcholine esterase by the neuromodulatory function of zinc, as all measurements were performed using the same buffer that contained physiological zinc concentrations. Thus, the differences must be based on developmentally acquired variances and are not due to acute alterations in zinc levels. In particular, we observed an increase in ACh signaling under zinc deficient conditions. This increase was seen both on the level of ACh induced currents and in the total number of cells responsive to ACh stimulation. More prominent, glutamatergic signaling was impaired under zinc deficient conditions. Again both a reduction in glutamate induced currents (both AMPAR and NMDAR currents) and a reduction in the total number of cells responding to glutamate stimulation were observed under zinc deficient conditions. In addition, GABAR currents and the fraction of cells displaying GABAR currents were significantly reduced. The results can be explained by an increase in the number of cholinergic synapses per neuron and a reduction of glutamatergic and GABAergic synapses per neuron and/or a strengthening of existing synapses. Indeed, we found a decrease of GABRA1 immunoreactive signals per dendrite as well as a reduction of GRIA3 levels per synapse supporting this model. We did not observe a significant increase in CHRNA3 signals. However, the CHRNA family alone consists of 10 members encoding for different subunits and more detailed analyses are needed in future studies. For example, on mRNA level, we observed mixed effects of neuronal differentiation under zinc deficient conditions on the cholinergic system. Here, the expression of many CHRNA family members was decreased under zinc deficient conditions, while the expression of CHRNA2 and 6 and CHRNB3 was increased.

Gene transcription was also found to be increased for some members of the glutamatergic and GABAergic system, such as GABRA1 and GRIA3 under zinc deficient conditions, where we detected a decrease of synaptic signals on protein level. It might be speculated that neuronal differentiation under zinc deficient conditions may result in a mislocalization of the proteins rather than decrease of total protein that cannot be overcome even upon increased gene transcription. Here, we only assessed synaptic protein concentrations. It is known, for example, that some synaptic glutamate receptors are dependent on a zinc sensitive PSD scaffold of SHANK2 and SHANK3 proteins for synaptic localization [10, 28].

Thus, it might be possible that zinc signals play a role in the establishment of neuronal subtype identity. Intriguingly, an excess of cholinergic neurons was found in the basal forebrain of autistic children [45] and nicotinic cholinergic antagonists have been reported beneficial in autism [46]. Although there are similarities in neuronal subtypes as previously discussed, here we used motor neurons as a model-neuron. It is possible that zinc signaling is regulated and involved in the differentiation of stem cells into other neuronal subtypes in alternative fashion.

Taken together, neurogenesis is an essential first step of the development of the CNS. The influence of zinc ions on cell differentiation and neuronal function can be complex and manifold and might span the whole spectrum from cell differentiation into mature neurons, followed by synaptogenesis, to synaptic pruning. In addition, programmed cell death is an important factor contributing to CNS development. Insufficient supply with zinc thereby might influence processes such as DNA replication, transcriptional control, mRNA translation, apoptosis, and microtubule stability [3, 47, 48]. Thus, zinc signaling and zinc levels need tight control by zinc transporters and buffering proteins. We conclude that sufficient supply with the essential trace metal zinc might be especially relevant during the time window of brain development and maternal zinc status might be a critical factor to secure healthy mental functioning. However, these findings might also be relevant to adult neurogenesis, as dietary zinc deprivation, zinc chelation, and depletion of vesicular zinc in ZnT3 knockout mice all lead to a significant decrease in hippocampal progenitor cell proliferation also in adult animal models [49].

## Supplementary Material

The Supplementary Material shows the quantitative evaluation of mRNA expression levels of selected zinc homeostasis and the zinc dependent SHANK genes under elevated zinc conditions (Figure S1) and the morphological analysis of motor neurons derived from iPS cells grown under control conditions and under altered zinc levels (Figure S2).

## References

[1] Morris D. R., Levenson C. W. (2013). Zinc regulation of transcriptional activity during retinoic acid-induced neuronal differentiation. *Journal of Nutritional Biochemistry*.

[2] Chowanadisai W., Graham D. M., Keen C. L., Rucker R. B., Messerli M. A. (2013). A zinc transporter gene required for development of the nervous system. *Communicative and Integrative Biology*.

[3] Levenson C. W., Morris D. (2011). Zinc and neurogenesis: making new neurons from development to adulthood. *Advances in Nutrition*.

[4] Marks P. A. (2010). Histone deacetylase inhibitors: a chemical genetics approach to understanding cellular functions. *Biochimica et Biophysica Acta (BBA)—Gene Regulatory Mechanisms*.

[5] Tapiero H., Tew K. D. (2003). Trace elements in human physiology and pathology: zinc and metallothioneins. *Biomedicine and Pharmacotherapy*.

[6] Freedman L. P., Luisi B. F. (1993). On the mechanism of DNA binding by nuclear hormone receptors: a structural and functional perspective. *Journal of Cellular Biochemistry*.

[7] O'Halloran T. V. (1993). Transition metals in control of gene expression. *Science*.

[8] Klug A., Schwabe J. W. (1995). Protein motifs 5. Zinc fingers. *The FASEB Journal*.

[9] Grabrucker A. M. (2013). Environmental factors in autism. *Frontiers in Psychiatry*.

[10] Grabrucker S., Jannetti L., Eckert M. (2014). Zinc deficiency dysregulates the synaptic ProSAP/Shank scaffold and might contribute to autism spectrum disorders. *Brain*.

[11] Grabrucker S., Boeckers T. M., Grabrucker A. M. (2016). Gender dependent evaluation of autism like behavior in mice exposed to prenatal zinc deficiency. *Frontiers in Behavioral Neuroscience*.

[12] Sutherland D. E. K., Stillman M. J. (2011). The ‘magic numbers’ of metallothionein. *Metallomics*.

[13] Floriańczyk B. (2011). Role of Zinc in nervous system cells. *Journal of Pre-Clinical and Clinical Research*.

[14] Gower-Winter S. D., Corniola R. S., Morgan T. J., Levenson C. W. (2013). Zinc deficiency regulates hippocampal gene expression and impairs neuronal differentiation. *Nutritional Neuroscience*.

[15] Linta L., Stockmann M., Kleinhans K. N. (2012). Rat embryonic fibroblasts improve reprogramming of human keratinocytes into induced pluripotent stem cells. *Stem Cells and Development*.

[16] Vitale A. M., Wolvetang E., MacKay-Sim A. (2011). Induced pluripotent stem cells: a new technology to study human diseases. *International Journal of Biochemistry and Cell Biology*.

[17] Takahashi K., Yamanaka S. (2006). Induction of pluripotent stem cells from mouse embryonic and adult fibroblast cultures by defined factors. *Cell*.

[18] Okano H., Yamanaka S. (2014). iPS cell technologies: significance and applications to CNS regeneration and disease. *Molecular Brain*.

[19] Hu B.-Y., Zhang S.-C. (2009). Differentiation of spinal motor neurons from pluripotent human stem cells. *Nature Protocols*.

[20] Hagmeyer S., Mangus K., Boeckers T. M., Grabrucker A. M. (2015). Effects of trace metal profiles characteristic for autism on synapses in cultured neurons. *Neural Plasticity*.

[21] Que E. L., Domaille D. W., Chang C. J. (2008). Metals in neurobiology: probing their chemistry and biology with molecular imaging. *Chemical Reviews*.

[22] Lichten L. A., Cousins R. J. (2009). Mammalian zinc transporters: nutritional and physiologic regulation. *Annual Review of Nutrition*.

[23] Cousins R. J., Liuzzi J. P., Lichten L. A. (2006). Mammalian zinc transport, trafficking, and signals. *The Journal of Biological Chemistry*.

[24] Bressler J. P., Olivi L., Cheong J. H., Kim Y., Maerten A., Bannon D. (2007). Metal transporters in intestine and brain: their involvement in metal-associated neurotoxicities. *Human & Experimental Toxicology*.

[25] Su R., Mei X., Wang Y., Zhang L. (2012). Regulation of zinc transporter 1 expression in dorsal horn of spinal cord after acute spinal cord injury of rats by dietary zinc. *Biological Trace Element Research*.

[26] Lindau M., Neher E. (1988). Patch-clamp techniques for time-resolved capacitance measurements in single cells. *Pflügers Archiv*.

[27] Stockmann M., Linta L., Föhr K. J. (2013). Developmental and functional nature of human iPSC derived motoneurons. *Stem Cell Reviews and Reports*.

[28] Grabrucker A. M. (2014). A role for synaptic zinc in ProSAP/Shank PSD scaffold malformation in autism spectrum disorders. *Developmental Neurobiology*.

[29] Tokheim A. M., Armitage I. M., Martin B. L. (2005). Antiserum specific for the intact isoform-3 of metallothionein. *Journal of Biochemical and Biophysical Methods*.

[30] Baron M. K., Boeckers T. M., Vaida B. (2006). An architectural framework that may lie at the core of the postsynaptic density. *Science*.

[31] Grabrucker A. M., Knight M. J., Proepper C. (2011). Concerted action of zinc and ProSAP/Shank in synaptogenesis and synapse maturation. *The EMBO Journal*.

[32] Raab M., Boeckers T. M., Neuhuber W. L. (2010). Proline-rich synapse-associated protein-1 and 2 (ProSAP1/Shank2 and ProSAP2/Shank3)-scaffolding proteins are also present in postsynaptic specializations of the peripheral nervous system. *Neuroscience*.

[33] Grabrucker S., Proepper C., Mangus K. (2014). The PSD protein ProSAP2/Shank3 displays synapto-nuclear shuttling which is deregulated in a schizophrenia-associated mutation. *Experimental Neurology*.

[34] Sandstead H. H., Fosmire G. J., Halas E. S., Strobel D., Duerre J., Kirchgessner M. (1977). Zinc: brain and behavioral development. *Trace Element Metabolsim in Man and Animals*.

[35] Vela G., Stark P., Socha M., Sauer A. K., Hagmeyer S., Grabrucker A. M. (2015). Zinc in gut-brain interaction in autism and neurological disorders. *Neural Plasticity*.

[36] Hagmeyer S., Haderspeck J. C., Grabrucker A. M. (2015). Behavioral impairments in animal models for zinc deficiency. *Frontiers in Behavioral Neuroscience*.

[37] Caulfield L., Black R. E., Ezzati M., Lopez A. D., Rodgers A., Murray C. J. L. (2004). Zinc deficiency. *Comparative Quantification of Health Risks: Global and Regional Burden of Disease Attributable to Selected Major Risk Factors*.

[38] Habela C. W., Song H., Ming G. L. (2015). Modeling synaptogenesis in schizophrenia and autism using human iPSC derived neurons. *Molecular and Cellular Neuroscience*.

[39] Volk L., Chiu S.-L., Sharma K., Huganir R. L. (2015). Glutamate synapses in human cognitive disorders. *Annual Review of Neuroscience*.

[40] Clegg M. S., Hanna L. A., Niles B. J., Momma T. Y., Keen C. L. (2005). Zinc deficiency-induced cell death. *IUBMB Life*.

[41] Corniola R. S., Tassabehji N. M., Hare J., Sharma G., Levenson C. W. (2008). Zinc deficiency impairs neuronal precursor cell proliferation and induces apoptosis via p53-mediated mechanisms. *Brain Research*.

[42] Chowanadisai W., Kelleher S. L., Lönnerdal B. (2005). Zinc deficiency is associated with increased brain zinc import and LIV-1 expression and decreased ZnT-1 expression in neonatal rats. *Journal of Nutrition*.

[43] Cousins R. J., Blanchard R. K., Popp M. P. (2003). A global view of the selectivity of zinc deprivation and excess on genes expressed in human THP-1 mononuclear cells. *Proceedings of the National Academy of Sciences of the United States of America*.

[44] Penkowa M., Giralt M., Thomsen P. S., Carrasco J., Hidalgo J. (2001). Zinc or copper deficiency-induced impaired inflammatory response to brain trauma may be caused by the concomitant metallothionein changes. *Journal of Neurotrauma*.

[45] Baumann M. L., Kemper T. L., Baumann M. L., Kemper T. L. (1994). Neuroanatomic observations of the brain in autism. *The Neurobiology of Autism*.

[46] Lippiello P. M. (2006). Nicotinic cholinergic antagonists: a novel approach for the treatment of autism. *Medical Hypotheses*.

[47] Pang W., Leng X., Lu H. (2013). Depletion of intracellular zinc induces apoptosis of cultured hippocampal neurons through suppression of ERK signaling pathway and activation of caspase-3. *Neuroscience Letters*.

[48] Adamo A. M., Oteiza P. I. (2010). Zinc deficiency and neurodevelopment: the case of neurons. *BioFactors*.

[49] Suh S. W., Won S. J., Hamby A. M. (2009). Decreased brain zinc availability reduces hippocampal neurogenesis in mice and rats. *Journal of Cerebral Blood Flow and Metabolism*.

