# Supplementary material for: Cellular Zinc Homeostasis Contributes to Neuronal Differentiation in Human Induced Pluripotent Stem Cells
Source: Neural Plast. 2016 May 9;2016:3760702. doi: 10.1155/2016/3760702 (PMC4876239; doi:10.1155/2016/3760702)
Supplement: Supplementary file 1 — The Supplementary Material shows the quantitative evaluation of mRNA expression levels of selected zinc homeostasis and the zinc dependent SHANK genes under elevated zinc conditions (Figure S1) and the morphological analysis of motor neurons derived from iPS cells grown under control conditions and under altered zinc levels (Figure S2). [file 3760702.f1.pdf]

Figure S1

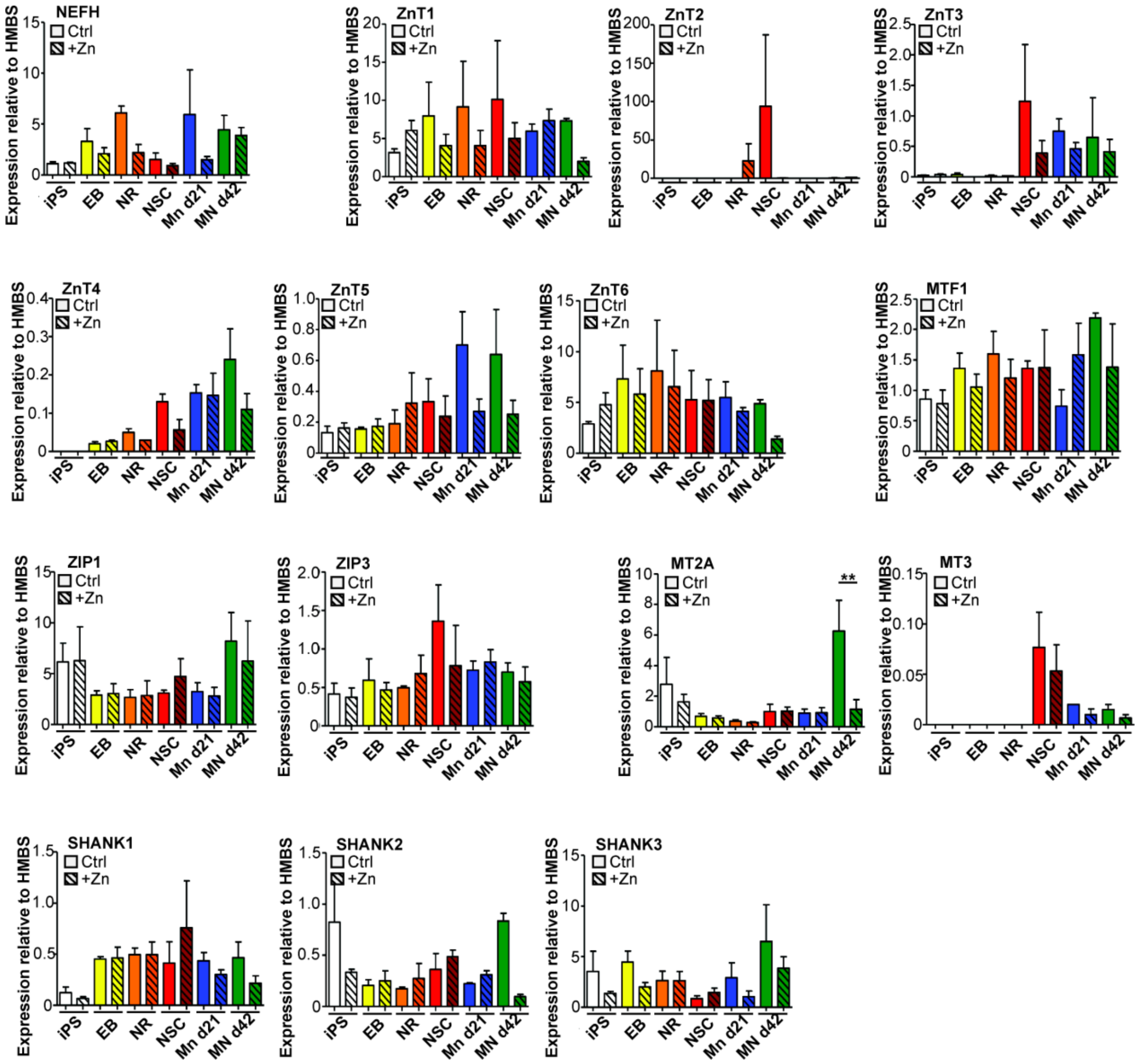

Figure S1: Quantitative evaluation of mRNA expression levels of selected zinc homeostasis and the zinc dependent SHANK genes under elevated zinc conditions (10 μM $ZnCl_2$). Analyses were performed in triplicates (n = 3). For statistical analysis, a 1-way ANOVA was used followed by Tukey's Multiple Comparison test. Although one-way ANOVA revealed significant differences for ZnT4 ($p = 0.0004$), MT2A ($p = 0.0053$), and MT3 ($p = 0.01$), of the post-hoc analyses comparing the treatment conditions within a differentiation stage only MT2A, $p = 0.0053$ (MN d42 Ctrl vs. +Zn, $p < 0.05$) showed significant changes. All other genes analyzed were not significantly altered.

Figure S2

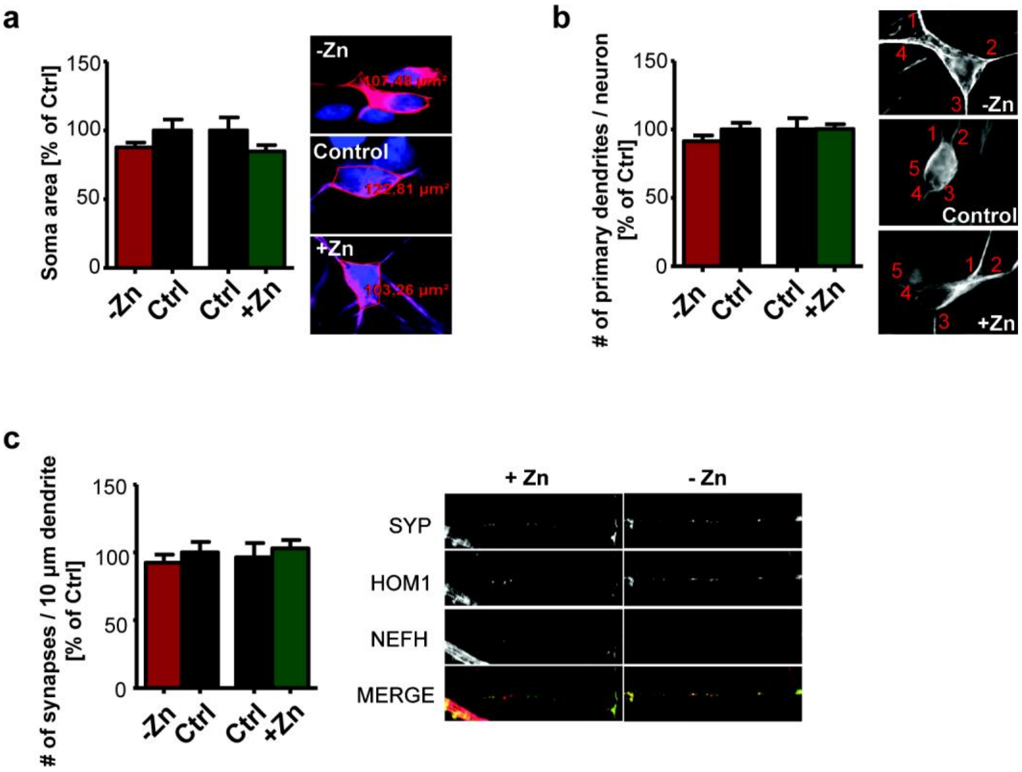

Figure S2: Morphological analysis of motor neurons derived from iPS cells grown under control and zinc supplemented conditions. A-C) The morphological analysis reveals no significant difference between controls and zinc supplemented or zinc depleted motor neurons regarding soma size (A) and the number of primary dendrites (B) assessed by NEFH staining and the number of synapses assessed by Homer1b/c & Synaptophysin immunoreactive fluorescent puncta per 10 μm dendrite length (C) (t-test, A,B: n = 42: $Ctrl_{-Zn}$, n= 29: -Zn, n = 24: $Ctrl_{+Zn}$, n = 111: +Zn; C: n = 60: $Ctrl_{-Zn}$ and -Zn, n = 28: $Ctrl_{+Zn}$, n = 72: +Zn). Right panel: Exemplary images of cells stained for Synaptophysin (SYP), Homer 1 (HOM1), and Neurofilament H (NEFH).
